# The conserved dileucine- and tyrosine-based motifs in MLV and MPMV envelope glycoproteins are both important to regulate a common Env intracellular trafficking

**DOI:** 10.1186/1742-4690-3-62

**Published:** 2006-09-15

**Authors:** Vincent Blot, Sandra Lopez-Vergès, Marie Breton, Claudine Pique, Clarisse Berlioz-Torrent, Marie-Pierre Grange

**Affiliations:** 1 Institut Cochin, DépartementBiologie Cellulaire, Paris, F-75014 France; 2 Institut Cochin, DépartementMaladies Infectieuses, Paris, F-75014 France; 3 Inserm, U567, Paris, F-75014 France; 4 CNRS, UMR 8104, Paris, F-75014 France; 5 Université Paris 5, Faculté de Médecine René Descartes, UMR3, Paris, F-75014 France; 6 Weill Medical College of Cornell, Biochemistry Dept, New York, NY10021 USA

## Abstract

**Background:**

Retrovirus particles emerge from the assembly of two structural protein components, Gag that is translated as a soluble protein in the cytoplasm of the host cells, and Env, a type I transmembrane protein. Because both components are translated in different intracellular compartments, elucidating the mechanisms of retrovirus assembly thus requires the study of their intracellular trafficking.

**Results:**

We used a CD25 (Tac) chimera-based approach to study the trafficking of Moloney murine leukemia virus and Mason-Pfizer monkey virus Env proteins. We found that the cytoplasmic tails (CTs) of both Env conserved two major signals that control a complex intracellular trafficking. A dileucine-based motif controls the sorting of the chimeras from the trans-Golgi network (TGN) toward endosomal compartments. Env proteins then follow a retrograde transport to the TGN due to the action of a tyrosine-based motif. Mutation of either motif induces the mis-localization of the chimeric proteins and both motifs are found to mediate interactions of the viral CTs with clathrin adaptors.

**Conclusion:**

This data reveals the unexpected complexity of the intracellular trafficking of retrovirus Env proteins that cycle between the TGN and endosomes. Given that Gag proteins hijack endosomal host proteins, our work suggests that the endosomal pathway may be used by retroviruses to ensure proper encountering of viral structural Gag and Env proteins in cells, an essential step of virus assembly.

## Background

Retroviruses are surrounded by a lipid envelope acquired by the virus from cellular membranes through a budding process. Anchored in this lipid envelope are the viral envelope glycoproteins (Env), which are heterodimers between a transmembrane subunit (TM) and a covalently or non-covalently attached extracellular subunit (named SU for surface). Both subunits emerge from the cleavage of a single type-1 transmembrane envelope glycoprotein precursor (for review on retrovirus structural protein synthesis, see [1].

The Gag proteins precursor, simply referred to here as Gag, is the only viral structural protein that is both necessary and sufficient to produce virus-like particles (VLPs) by budding into the extracellular medium, even in the absence of Env [2,3]. However, VLPs devoid of Env are non infectious since Env glycoproteins are necessary for the attachment of the virions to their receptor(s) and subsequent fusion of viral and target cell membranes leading to virus entry. The Env precursor is co-translationally anchored in the membrane of the endoplasmic reticulum and then follows the trafficking of transmembrane and soluble proteins along the secretory pathway. By contrast, Gag is synthesized by free ribosomes in the cytosol, before being able to bind to internal membranes through signals in its amino-terminus. Given that both structural components are being translated in different subcellular compartments, some specific mechanisms must account for their encounter at the site of virus assembly and budding.

Studying the precise steps of the intracellular trafficking of envelope glycoproteins should then bring some understanding as to how they encounter Gag in cells. In the case of human immunodeficiency virus (HIV) Env, it has been shown that the cytoplasmic tail (CT) of the TM subunit contains several motifs that regulate Env trafficking. A tyrosine-based motif (YxxΦ where Φ is a bulky hydrophobic amino-acid) has been implicated in Env endocytosis after its arrival at the cell surface by mediating interaction with the AP-2 clathrin adaptor complexes [4-7]. A dileucine-based motif (consensus sequence LL or LΦ) has also been shown to control some post-Golgi trafficking step by recruiting the AP-1 adaptor complexes [5,8]. Finally, HIV Env is also able to undergo a retrograde endosome to trans-Golgi network (TGN) route through the interaction of a diaromatic YW motif, located in the cytoplasmic domain of Env, with the TIP47 protein [9].

The intracellular transport of HIV Env glycoproteins has been extensively examined, however little is known about the trafficking of envelope glycoproteins of retroviruses that do not belong to the lentivirus genus. The cytoplasmic tails of human T-cell leukemia virus (HTLV) and Moloney murine leukemia virus (MLV) Env possess a tyrosine-based motif that is able to target them to the basolateral membrane of polarized MDCK cells [10]. Dileucine- and tyrosine-based motifs in the CT of bovine leukemia virus (BLV) Env are responsible for low surface expression of Env, although the details of Env intracellular trafficking were not elucidated [11]. We have shown in a previous study that engrafting the CTs of different retrovirus Env to the carboxy-terminus of the CD25 reporter molecule leads to specific intracellular trafficking pathways of the resulting chimeras [12]. Indeed, HTLV, BLV and Rous sarcoma virus (RSV) CD25 chimeras are endocytosed after reaching the cell surface, whereas chimeras containing either MLV or Mason-Pfizer monkey virus (MPMV) CT appeared mainly retained inside the cells in a Rab6-positive Golgi or post-Golgi compartment.

In this study, we aimed to precisely define the intracellular routes followed by MLV and MPMV envelope glycoproteins. Using the same CD25 chimera-based approach, we found that these proteins accumulated in the TGN as a result of a dynamic transport involving a retrograde route from endosomes to the TGN. A membrane proximal dileucine-based motif and a more distal tyrosine-based motif conserved between both CTs governed this peculiar trafficking. The dileucine-based motif is implicated in the sorting of the chimeras at the level of the TGN, whereas the tyrosine-based motif is required in the retrograde transport step. We also documented that both motif mediate *in vitro *interaction with clathrin adaptors, linking their functional role in Env trafficking with their capacity to physically interact with cellular trafficking machineries.

## Results

### CD25-MuLV and CD25-MPMV chimera accumulated in the TGN

We have previously shown that engrafting the cytoplasmic tail of either MLV or MPMV envelope glycoprotein to the carboxyl-terminus of the CD25 protein induced the intracellular retention of the resulting chimeras [12]. Both chimeras colocalized at steady state with the small GTPase Rab6, a protein distributed between the Golgi apparatus and the TGN [13,14].

**Figure 1:**
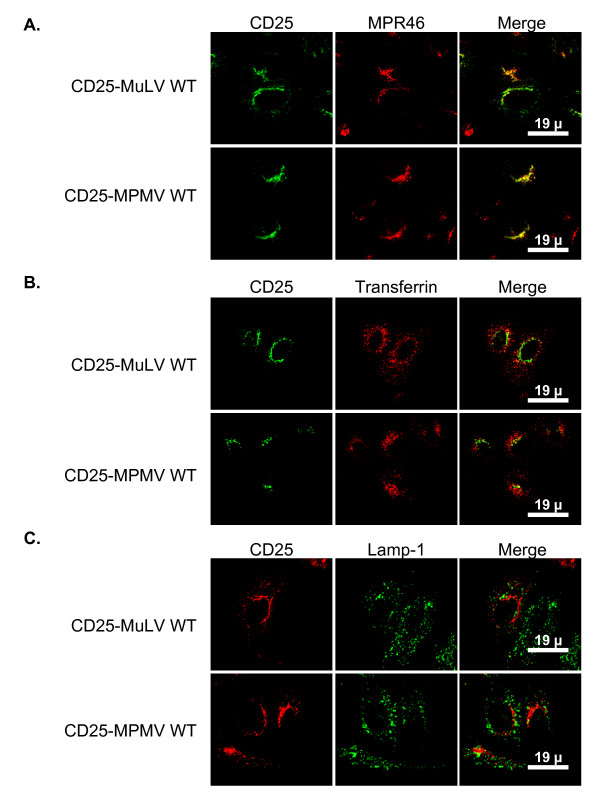
**CD25-MLV and CD25-MPMV accumulate in the TGN**. Forty-eight hours after transfection with the appropriate chimera cDNA, cells were treated with cycloheximide for 3 hours prior to fixation and staining. **A. **Co-staining of CD25 chimeras and Mannose 6-phosphate receptor of 46 kDa (MPR46), a protein that accumulates in the TGN at steady state. **B. **Co-staining of CD25 chimeras and internalized Cy3-conjugated tranferrin revealing the early/recycling endosomes. **C. **Co-staining of CD25 chimeras and Lamp1, a protein resident of the lysosomes.

To define more precisely the intracellular site of accumulation of the chimeras, we treated transiently transfected HeLa cells with cycloheximide, which acted by preventing new synthesis of proteins. CD25-MuLV and CD25-MPMV chimeras appeared then mainly concentrated in a tubular-shaped perinuclear compartment as well as in dots dispersed throughout the cytoplasm (figure 1, CD25 panels) whereas the control CD25 protein accumulated at the cell surface (data not shown and [12]).

We then compared the distribution of the chimeras with those of different intracellular markers: the Mannose-6-phosphate receptor of 46kDa (MPR46) that cycles between the TGN and late endosomes and is mainly localized in the TGN at steady state [15], internalized cyanin3-conjugated transferrin that reveals the general early and recycling endosomal pathway and Lamp1, a marker of lysosomes [16]. CD25-MLV and CD25-MPMV did not colocalize with either endocytosed transferrin or Lamp1, indicating that they do not accumulate in the endocytic pathway (figure 1B and 1C). By contrast, both proteins showed extensive colocalization with MPR46 revealing that their intracellular compartment of retention is the TGN (figure 1A).

### A dileucine- and a tyrosine-based motifs are both required for the TGN localization of CD25-MuLV and CD25-MPMV chimeras

**Figure 2:**
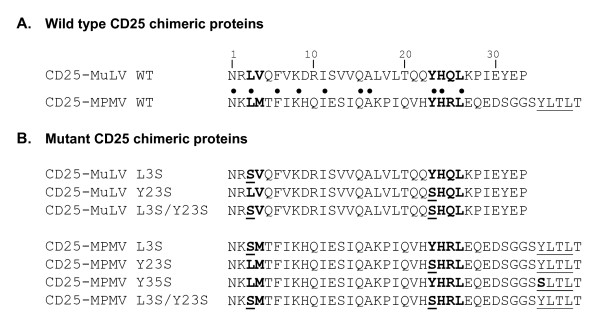
**Sequences of wild type and mutant MLV and MPMV cytoplasmic tails**. **A. **The 10 amino acids conserved between MLV and MPMV cytoplasmic tails (CT) are noted ● Bold letters indicate the position of the conserved dileucine- and tyrosine-based motifs, whereas underlined letters indicate the position of the extra tyrosine-based motif in MPMV CT. **B. **Sequences of the mutated CD25 chimeras that we used in this study. The mutants are named CD25-retrovirus X amino acid position Z, where X and Z are the wild-type and mutant amino-acids, respectively. The amino-acid position 1 corresponds to the first residue of the corresponding viral CT.

To define the motifs in MLV and MPMV cytoplasmic tails important for this peculiar localization, we compared their primary sequences (figure 2A). The two sequences shared 10 amino acids conserved in position, amongst which two clusters fit potential conventional sorting signals: the dileucine-based motifs ^3^LV^4^/^3^LM^4 ^and the tyrosine-based motif ^23^YHQL^26^/^23^YHRL^26 ^in MLV and MPMV sequences respectively (where 1 is the position of the first amino-acid in each viral cytoplasmic tail). MPMV cytoplasmic tail possesses a second tyrosine-based motif (^35^YLTL^38^) that is not conserved in the MLV cytoplasmic domain.

**Figure 3:**
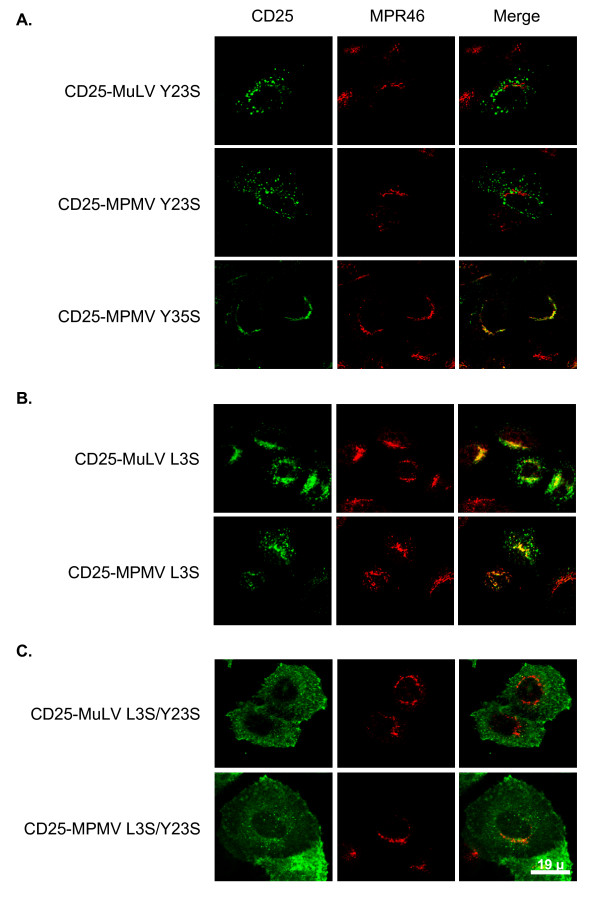
**Mutation of either the dileucine- or the tyrosine-based motifs affect the TGN localization of CD25 chimeras**. Forty-eight hours after transfection with the appropriate chimera cDNA, cells were treated with cycloheximide for 3 hours prior to fixation and permeabilization. Co-stainings of MPR46 and chimeras bearing either (**A**) the Y23S or the Y35S mutation, (**B**) the L3S mutation, or (**C**) both L3S and Y23S mutations.

To investigate the implication of these putative sorting motifs in the trafficking of the chimeras, we produced a diversity of point mutations in the cytoplasmic tails by site-directed mutagenesis (figure 2B). We then analyzed the effects of these mutations on the intracellular localization of the resulting mutated chimeras. Mutation of the tyrosine 23 to serine in either MLV and MPMV CT provoked a relocalization of the chimeras to peripheral dots dispersed throughout the cytoplasm that do not colocalize with MPR46 (figure 3A). By contrast, mutation of the distal ^35^YLTL^38 ^tyrosine-based motif in MPMV cytoplasmic tail had no effects (figure 3A lower panels). Changing the leucine 3 into a serine resulted in a partial shift of the localization of the chimeras from the TGN to peripheral dots and the mutated chimeras still colocalized to some extent with MPR46 (figure 3B). Finally, MLV and MPMV chimeras mutated on both leucine 3 and tyrosine 23 mainly accumulated at the plasma membrane (figure 3C), thus behaving as the control CD25.

Thus, extensive localization of the CD25-MLV and the CD25-MPMV chimeras in the TGN required both the dileucine-based motif in position 3 and the tyrosine-based motif in position 23. By contrast, the tyrosine-based motif in position 35 of the MPMV cytoplasmic tail does not play a significant role in the TGN localization of the protein.

### CD25-MLV and CD25-MPMV with mutated dileucine- or tyrosine-based motifs accumulate in different endocytic compartments

We then assess whether the changes in localization of the CD25-MLV and CD25-MPMV chimeras that we observed after mutating either the dileucine- or the tyrosine-based motif revealed a relocalization of the protein in endocytic compartments. We used internalized transferrin as a marker of early/recycling endosomes, Lamp1 as a marker of lysosomes and dextran internalized for 30 minutes and chased for an equivalent amount of time to reveal late endosomal compartments.

**Figure 4:**
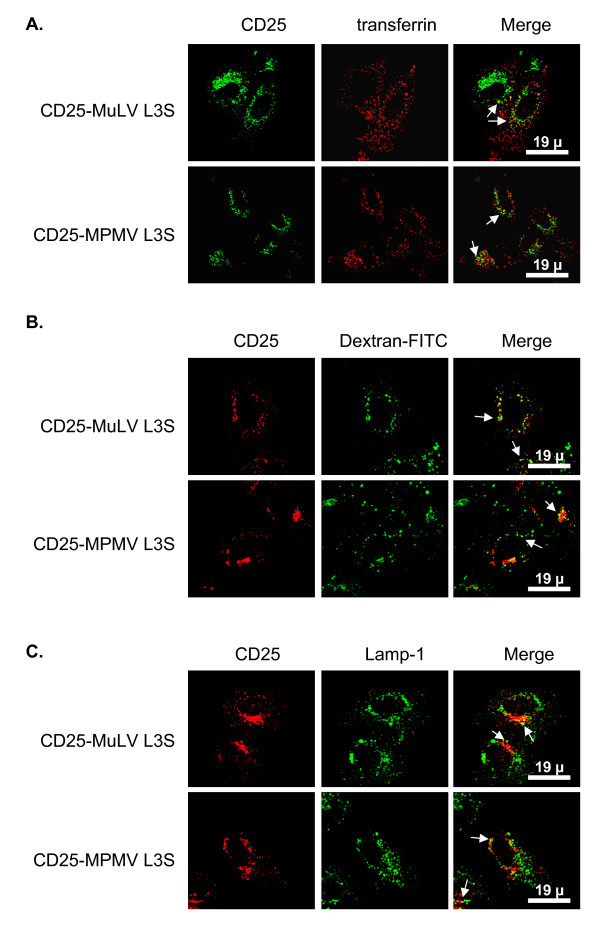
**Chimeras bearing the L3S mutation are relocated throughout the endosomal pathway**. Forty-eight hours after transfection with the L3S mutant chimeras cDNA, HeLa cells were treated with cycloheximide for 3 hour prior to fixation and permeabilization. **A. **Co-staining of L3S mutant chimeras and internalized Cy3-conjugated transferrin revealing the early/recycling endosomes.**B. **Cells were allowed to take up FITC-conjugated dextran for 30 min. Cells were then extensively washed, and dextran was chased for another 30 min prior to fixation and CD25 staining. FITC-dextran thus revealed some late endosomal compartment. **C. **Co-staining of CD25 chimeras and Lamp1, a protein resident of the lysosomes.

**Figure 5:**
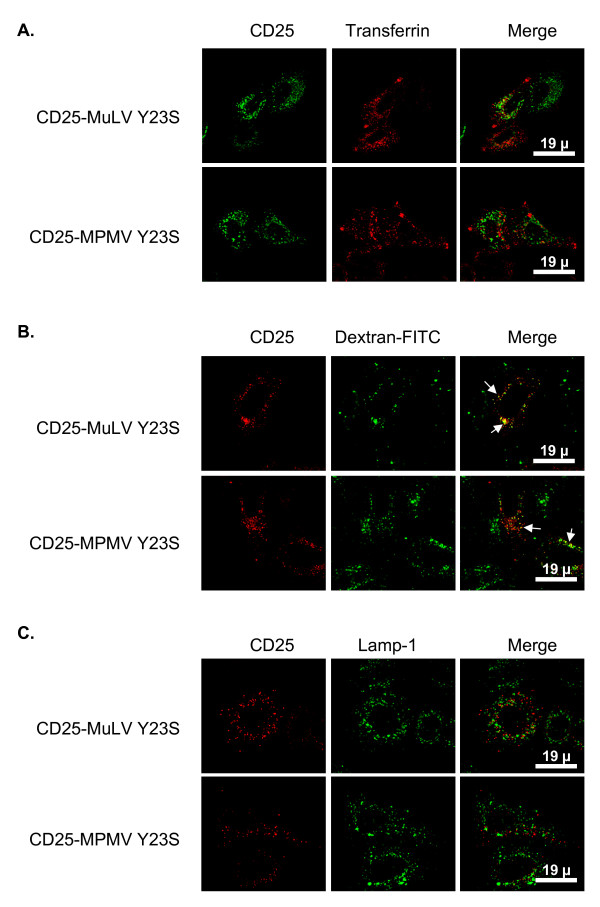
**Chimeras bearing the Y23S mutation are mainly relocated to a late endosomal compartment**. Forty-eight hours after transfection with the Y23S mutant chimeras cDNA, HeLa cells were treated with cycloheximide for 3 hour prior to fixation and permeabilization. **A. **Co-staining of Y23S mutant chimeras and internalized Cy3-conjugated transferrin revealing the early/recycling endosomes.**B. **Before fixation, cells were allowed to take up FITC-conjugated dextran for 30 min. The cells were then extensively washed, and dextran was chased for another 30 min prior to fixation thus accumulating in late endosomal compartments. **C. **Co-staining of CD25 chimeras and Lamp1, a protein resident of the lysosomes.

Chimeras with mutations in the dileucine-based motif showed partial colocalization with the three markers of the endosomal pathway (figure 4A, 4B and 4C, arrows). Colocalization of chimeras with Lamp1, however, is weaker than with endocytosed transferrin or dextran. Thus, the fraction of L3S mutated chimeras that is delocalized from the TGN is redistributed throughout the endosomal pathway. By contrast, chimeras bearing the Y23S mutation did not colocalize with either transferrin or Lamp1 (figure 5A and 5C), indicating that they are absent from early/recycling endosomes or lysosomes. However, these mutant proteins did colocalize to some extent with internalized and chased dextran (figure 5B, arrows). Thus, mutation of the tyrosine-based motif in position 23 induced the relocalization of both CD25-MLV and CD25-MPMV chimeras in non well-defined late endosomal compartments.

### Internalization of chimeras from the plasma membrane is mainly driven by the tyrosine-based motif in position 23

That the chimeras are mainly detected in intracellular sites at steady state could either reflect an active retention of the proteins within the cells or their slow recycling to the plasma membrane followed by their rapid internalization. We thus wanted to determine whether the chimeras could be endocytosed from the plasma membrane. To that extent, we compared the abilities of the different WT and mutant chimeras to allow uptake of monoclonal anti-CD25 antibody. Transiently-transfected HeLa cells were then incubated for 30 min at 4°C with anti-CD25 antibody and shifted or not at 37°C for 30 additional minutes. For each chimera, we then compared the amount of anti-CD25 antibody remaining at the cell surface after 30 minutes at 37°C relative to the amount of anti-CD25 at the cell surface at time 0.

**Figure 6:**
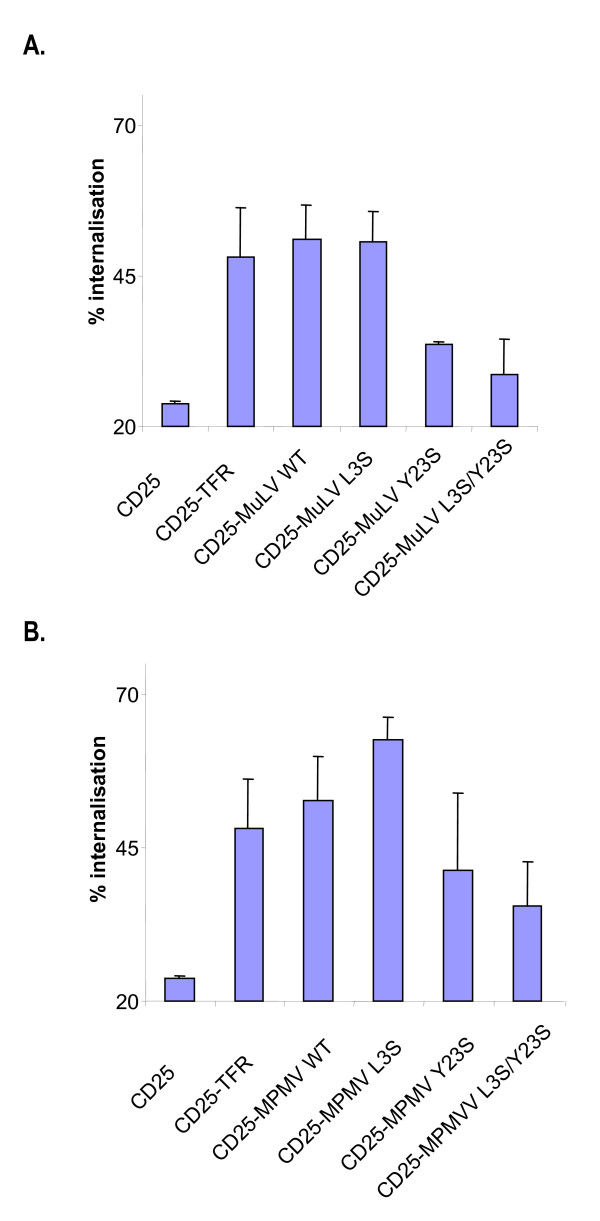
**Effects of the L3S and/or Y23S mutations on the chimeras ability to be retrieved from the plasma membrane**. HeLa cells were cotransfected with GFP vector and with the appropriate **A **MLV or **B **MPMV chimera cDNA. Cells were then incubated for 1 hour at 4C with anti-CD25 antibody before being either shifted for 30 min at 37°C or not. Anti-CD25 stainings were revealed using phycoerythrine-conjugated secondary antibodies. Stained cells were analyzed using flow cytometry excluding the none transfected GFP-negative cells. We then plotted the percentage of internalization as the ratio between the CD25-associated fluorescence that disappeared during the 30 min uptake at 37°C and the CD25-associated fluorescence at time 0. CD25 is the reference protein without any viral cytoplasmic tail (negative control) and CD25-TFR is the CD25 reference protein in the cytoplasmic tail of which the well described YTRF endocytosis motif of the transferrin receptor has been inserted (positive control).

After 30 minutes, approximately 50% of bound anti-CD25 antibody was internalized in cells expressing either CD25-MLV or CD25-MPMV chimeras. This is similar to the amount of CD25 internalized in cells expressing CD25-TFR, a control chimera containing the well defined YRTF endocytic signal of the transferrin receptor (figure 6A and 6B). By contrast, the CD25 control protein that lacks specific internalization signals or viral cytoplasmic tail does not allow measurable uptake of anti-CD25 antibody. This indicates that viral cytoplasmic tails in CD25-MLV and CD25-MPMV chimeras contain specific internalization signals.

Mutation of the dileucine-based motifs in MLV or MPMV chimera did not impair the capacity of the proteins to mediate specific uptake anti-CD25 antibody (fig 6A and 6B; L3S). By contrast, chimeras bearing the Y23S mutation had a decreased ability to allow anti-CD25 antibody retrieval from the cell surface (figure 6A and 6B). Chimeras bearing both L3S and Y23S mutations behave like the single Y23S mutant indicating that the lack of detectable effects of the single L3S mutation was not due to redundancy with the Y23 tyrosine-based motif.

Altogether, these results indicate that CD25-MLV and CD25-MPMV chimeras are internalized from the plasma membrane, and that the tyrosine-based motif in position 23 acts as their main endocytosis signal.

### The tyrosine-based motif in position 23 drives a retrograde transport step toward the TGN

The steady state TGN localization of proteins like MPRs, furin or TGN38 is the results of a complex trafficking involving a retrograde transport from endosomes to the TGN [15,17]. We thus assessed the capacity of MLV and MPMV cytoplasmic tails to target the chimeras to the TGN following their internalization in endosomes.

**Figure 7:**
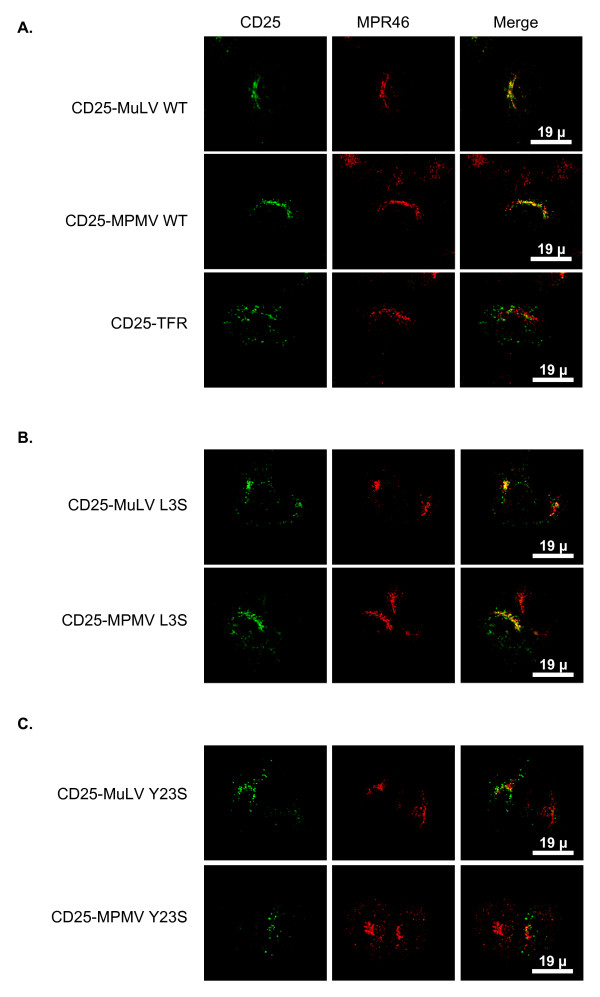
**The tyrosine-based motif in position 23 allows the chimera to follow a retrograde route from endosomes to TGN**. HeLa cells were transfected with **A **wild type, **B **L3S mutated or **C **Y23S mutated chimeras. Forty-eight hours after transfection, cells were treated with cycloheximide for 2 h. Chimeras present on cell surface were stained with the anti-CD25 antibody at 4°C for 1 hour and cells were then shifted at 37°C for another hour. After fixation, internalized anti-CD25 was revealed using FITC-conjugated secondary antibodies, and MPR46 was revealed as in figure 1.

One hour after their internalization from the cell surface, anti-CD25 antibodies taken up by either the CD25-MLV or CD25-MPMV chimera were found concentrated in a perinuclear region of the cells (figure 7A). Both chimeras then extensively colocalized with MPR46, indicating that they reached the TGN (figure 7A). By contrast, anti-CD25 taken up by the control CD25-TFR construct that follows the recycling pathway of the transferrin receptor did not colocalize with MPR46 (figure 7A), indicating that both MLV and MPMV cytoplasmic tails contain specific information capable of driving their retrograde transport to the TGN.

Mutation of the dileucine motif in position 3 did not drastically affect the capacity of the chimeras to be targeted to the TGN following internalization (Figure 7B). By contrast, chimeras mutated in the tyrosine-based motif in position 23 appeared localized in dispersed dots throughout the cytoplasm after their internalization. No colocalization was then apparent with MPR46 (Figure 7C).

Altogether, these data indicate that the TGN localization of the MLV and MPMV chimeras is the result of a complex trafficking involving retrieval of these proteins from endosomal compartments towards the TGN. This last step is driven by the tyrosine-based motif in position 23 that is conserved between both retroviruses.

### MLV cytoplasmic tail interacts with adaptor protein complexes (AP) 1, 2 and 3

**Figure 8:**
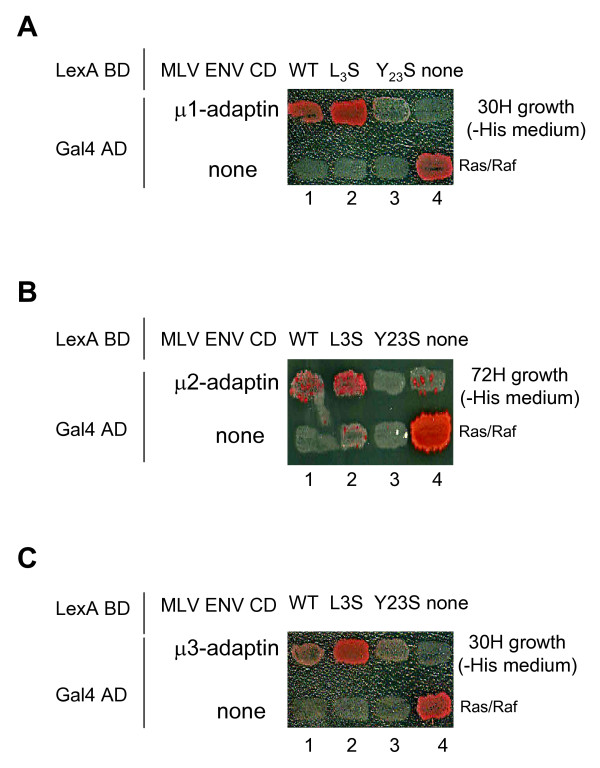
**Interaction of the MLV TM-CD with μ1, μ2 and μ3 subunits in the yeast two-hybrid system**. The yeast reporter strain L40 was co-transformed with plasmids encoding Gal4 AD-μ1, μ2 and μ3-adaptin (**A**, **B **and **C**, respectively), and plasmids encoding lexA BD fused to either the wild type (WT) or mutated (L3S, Y23S) cytoplasmic tail of MLV. Cotransformants were analyzed for histidine auxotrophy. They were patched on medium with histidine and then replica-plated on medium without histidine (- His medium). Growth in the absence of histidine indicates interaction between hybrid proteins. The positive control was the interaction between Ras and Raf proteins which bind to each other efficiently (lane 4, lower patch in panels A, B, C). Binding specificity was verified by the absence of interaction between the retroviral cytoplasmic tails (WT, L3S and Y23S) and the Gal4AD alone (none).

To better understand the molecular basis of the intracellular sorting of the viral chimeras, we assessed the ability of the viral CT to physically interact with components of the adaptor protein complexes AP-1, AP-2 and AP-3 in a yeast two-hybrid assay. Because we have shown that both MLV and MPMV Env share the same trafficking, we decided to restrict our biochemical analysis to one virus. Thus, MLV CT was fused to the N-terminus of the LexA binding domain (BD), whereas the μ1, γ and β1 chains of AP1, the μ2, α and β2 chains of AP2 and the μ3, δ and β3 chains of AP3 were fused to the Gal4 activation domain (AD). MLV CT did not interact with γ or β1 subunits of AP1, α or β2 subunits of AP2, or δ and β3 subunits of AP3 in yeast two-hybrid system (data not shown). By contrast, MLV CT bound to μ1, μ2 and μ3 medium chains as indicated by the expression of the HIS3 reporter gene, which allows cell growth in the absence of histidine (figure 8A, 8B and 8C). However, interaction with μ2 only appeared after 72 hours growth (figure 8B), whereas interaction with μ1 and μ3 were present after 30 hours growth (figure 8A and 8C), indicating that binding to μ2 was weaker than the other interactions.

Mutation of the tyrosine in position 23 completely abolished interaction of the MLV CT with all three μ1, μ2 and μ3 chains of AP complexes (figure 8A, 8B and 8C). On the contrary, mutation of the leucine in position 3 did not affect interaction with any of the μ chains (figure 8A, 8B and 8C). These results therefore indicate the tyrosine 23 is critical for binding of the MLV cytoplasmic tail to the isolated μ subunits, and further demonstrate the specificity of these interactions.

**Figure 9:**
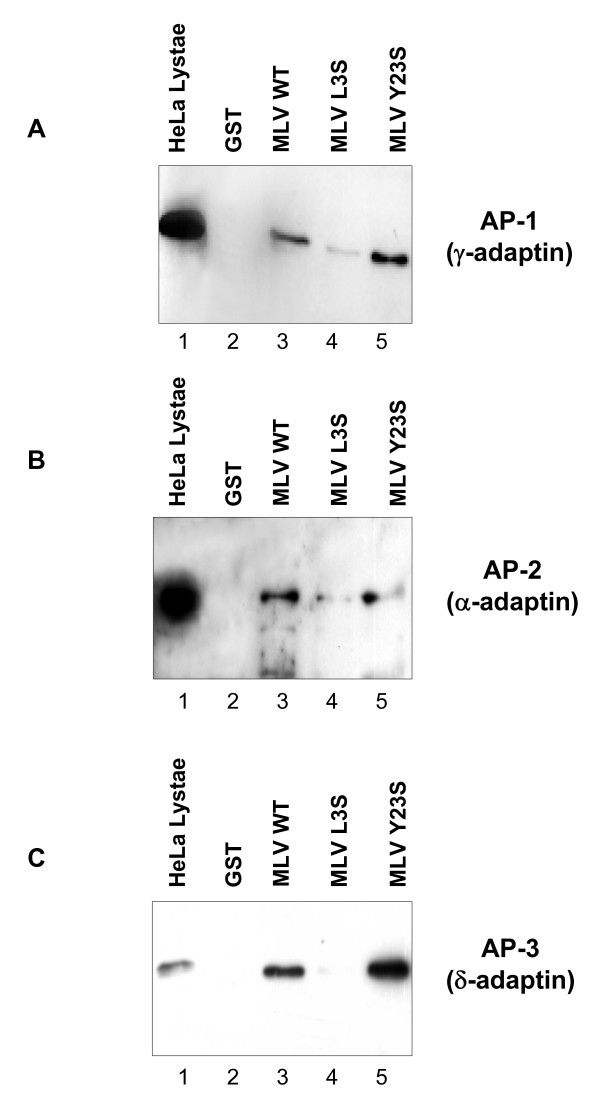
**Interaction between GST fusions of WT or mutated MLV cytoplasmic tail and AP-1, AP-2 and AP-3 complexes**. Identical quantities of GST (5 μg) (lanes 2, panels **A-C**), GST-MLV (lanes 3), GST-MLV-L3S (lanes 4,), GST-MLV-Y23S (lanes 5) were incubated with HeLa cell lysates (2.5 × 10^6 ^cells). The binding of AP-1, AP-2 and AP-3 complexes to GST fusion proteins was revealed by Western blotting with anti-γ adaptin mAb (panel **A**), anti-α adaptin mAb (panel **B**) and anti-δ adaptin mAb (panel **C**). The positions of the α-adaptin (*Mr*~100,000), γ-adaptin (*Mr*~104,000) and δ-adaptin (*Mr*~90,000) are indicated in the crude cell lysate from 10^6 ^cells (lanes 1).

We then examined whether a GST fusion of the MLV CT was able to recruit the whole preformed AP complexes from HeLa cells lysates. AP1, AP2 and AP3 complexes were revealed using antibodies to γ-adaptin, α-adaptin and δ-adaptin, respectively. Immunoblot analysis of the cellular proteins retained on GST-MLV beads indicated that AP1, AP2 and AP3 bound specifically to the viral cytoplasmic tail (figure 9A, 9B and 9C). Mutation of either the tyrosine 23 or the leucine 3 affected the binding of the resulting GST-MLV to the AP2 complex (figure 9B). Interestingly, mutating the leucine 3 strongly affected the binding to AP1 and AP3, whereas mutation of the tyrosine 23 had no effect (figure 9A and 9C).

We thus showed that MLV cytoplasmic tail interacts not only with the μ chains of clathrin adaptors type 1, 2 and 3, but also associates with the AP complexes from cell lysates. Optimal interaction with AP2 requires both the dileucine- and the tyrosine-based motif in position 3 and 23, respectively. On the other hand, interactions with AP1 and AP3 complexes depend only on the dileucine-based motif.

## Discussion

In this study, we analyzed the intracellular trafficking of two oncoretroviral envelope proteins, these of MLV and MPMV retroviruses. Their peculiar trafficking resulted in the dynamic intracellular retention of the proteins in the TGN and was driven by the association of two conventional sorting signals conserved in position between the two envelope glycoprotein cytoplasmic tails: a membrane proximal dileucine-based motif (^3^LV^4^/^3^LM^4 ^in MLV and MPMV sequences respectively) and a more distal tyrosine-based motif (^23^YHQL^26^/^23^YHRL^26^).

To evaluate the roles of the CT of MLV and MPMV envelope glycoproteins in regulating their trafficking, we used an approach based on the study of chimeras between the whole CD25 chain and the cytoplasmic tail of retroviral Env proteins. Study of CD25 chimeras is a broadly used approach to assess the role of the cytoplasmic tail of different cellular proteins in their trafficking [17-19]. We and others have used this approach to study the trafficking of different retroviral glycoproteins [5,9,11,12]. Using CD25 chimeras permitted us to conduct a comparative work and to avoid complications inherent to the use of native viral envelope glycoproteins such, as shedding of the SU subunit in the extracellular medium or cytopathogenic effects due to envelope induced cell-cell fusion. Importantly, we previously demonstrated that native viral glycoproteins displayed the same intracellular trafficking as their chimeras counterparts, thus legitimazing the use of this approach [8,9,12].

We previously showed that CD25-MLV and CD25-MPMV chimera appeared retained in an intracellular tubular-shaped perinuclear compartment [12]. We now show that this compartment is distinct from the early/recycling endosomes or lysosomes and is enriched with the MPR46 protein. MPR46 mediates the transport of lysosomal enzymes from the TGN to endosomal prelysosomal compartments. After delivery of its cargo in acidic compartments, MPR returns to the TGN [20]. At steady state, MPR46 is found in the TGN, although a fraction may be found in endosomes [21,22]. We therefore concluded that the majority of CD25-MLV and CD25-MPMV localized in the TGN.

The TGN localization of both chimeras was dependent on the integrity of two sorting motifs. Mutating the dileucine-based motif in position 3 resulted in a partial delocalization of the chimeras throughout the endosomal pathway, without affecting their capacity to follow the retrograde route to the TGN when internalized from the plasma membrane. By contrast, mutation of the tyrosine-based motif in position 23 totally abolished the TGN localization of the chimeras, as well as their ability to be retrieved from endosomes to the TGN. The Y23S mutated chimeras then appeared accumulated in an endosomal compartment. Because this compartment was stained after internalization of dextran followed by a chase and was distinct from the early/recycling endosomes and lysosomes, we conclude that it must represent some late endosomal compartment. This compartment, however, did not contain CD63, a typical marker for late endosmes/multivesicular bodies (data not shown) and its exact nature remains to be identified. Regardless, these data allow us to propose the following model for the complex intracellular trafficking of the CD25-MLV and CD25-MPMV chimeras:

While exiting the biosynthetic pathway at the TGN level, the chimeric proteins are sorted to a specific late endosomal compartment. This sorting step involves the dileucine-based motif and mutation of this motif results in misrouting the proteins throughout the endosomal pathway (Table 1). Alternatively, Env proteins can also reach the late endosomal compartment after internalization from the plasma membrane. Once the chimeras reach the specific late endosomal compartment, their tyrosine-based motif in position 23 mediates their trafficking in a retrograde pathway up to the TGN. If the tyrosine-based motif is mutated, the chimeras accumulate in the late endosomal compartment (Table 1). On the contrary, the wild type chimeras continuously cycle between endosomes and the TGN. The fact that chimeric proteins appear accumulated in the TGN at steady state indicates that the limiting step of their trafficking is the sorting event at the TGN level. Lastly, chimeras that make it to the plasma membrane are being internalized and delivered back to the TGN, both steps implicating the tyrosine-based motif in position 23 (Table 1).

**Table 1:** Summary of the roles of the motifs in MLV and MPMV Env CT in subcellular Env trafficking.

MOTIF (MLV/MPMV)	ROLE IN TRAFFICKING	EFFECT OF MUTATION
^23^YHQL^26^/^23^YHRL^26^	Endocytosis	Increase plasma membrane localization
^23^YHQL^26^/^23^YHRL^26^	TGN retrieval	Delocalization in unidentified late-endocytic compartments
^3^LV^4^/^3^LM^4^	Sorting from TGN	Delocalization in the endosomal pathway

This model is reinforced by the biochemical study we conducted on the MLV tail. Indeed, GST pull down assays confirmed that the dileucine-based motif is important for interaction with AP1 and AP3 complexes that are implicated in sorting cargos at the TGN level (review in [23]), whereas the tyrosine-based motif is somehow dispensable for this process. On the contrary both dileucine- and tyrosine-based motifs are important for the optimal recruitment of the AP2 complexes that function in endocytosis [24]. However, the direct interaction we detected in yeast two-hybrid between MLV cytoplasmic tail and the μ2 chain of AP2 appeared weaker than interaction with μ1 or μ3. This is consistent with our findings that trafficking of CD25-MLV chimeras is mainly restricted between intracellular compartments and that the MLV tyrosine-based signal is not optimized for the binding to the AP2 complexes. These findings nevertheless indicate that when the chimeras eventually reach the plasma membrane, they can be cleared from cell surface following endocytosis.

Our biochemical data does not elucidate how the tyrosine-based motif in position 23 mediates the retrograde route of the CD25-MLV and CD25-MPMV chimeras from late endosomal compartment to the TGN. One retrograde transport pathway to TGN involves the AP1 clathrin adaptor [25,26]. In our yeast two-hybrid experiments, we elucidated an interaction between the μ1 chain of AP1 and MLV cytoplasmic tail. This interaction depended on the integrity of the tyrosine-based motif in position 23. In our GST pull down assay, however, the tyrosine 23 appears dispensable for the interaction with AP1 complexes. These contrasting results might indicate that, although the tyrosine-based motif is capable to bind the isolated μ chains of AP complexes in the yeast two-hybrid system, it might not be able to mediate the interaction with the whole AP1 complex. Alternatively, it is also possible that the interaction of the MLV and MPMV Env CTs with AP1 complexes during the retrograde transport might involve additional informations, like other determinants located in the Env CTs. Another possibility is that the tyrosine-based motif functions in the retrograde transport by recruiting other adaptors than AP1. Indeed, different retrograde pathways have been described for the furin and MPRs proteins. Retrograde transport of the furin protein involves an interaction of an acid cluster of amino-acids with the adaptor protein PACS1 [27,28] while retrograde transport of the MPRs requires either interaction of a motif constituted by two successive aromatic amino-acids (MPR46) or by prolines (MPR300) with the TIP47 adaptor [29,30]. None of these kinds of motifs can be found in either MLV or MPMV cytoplasmic tails suggesting that these viral proteins follow a different retrograde route using an unknown mechanism or use new motifs to interact with these adaptors. Formal demonstration of this would however require more biochemical analysis to directly test the interaction of MLV and MPMV cytoplasmic tails with PACS1 and TIP47.

Interestingly, it has been demonstrated that Epsin R can recruit clathrin either directly or through AP1 [31-34]. This recruitment is important for an AP1-independent retrograde pathway followed by TGN38 and MPR300 [35]. Because it has proposed that Espin R may function as a cargo adaptor [36], further studies should also assess the putative relationship between Epsin R and the retrograde transport mediated by MLV and MPMV cytoplasmic tails.

The dileucine 3- and tyrosine 23-based motifs in MLV and MPMV cytoplasmic tails are very similar to each other in term of sequence and are conserved in position (Fig 2). Nevertheless, MLV and MPMV infect different hosts, belong to two different retrovirus genuses and appear highly divergent on a phylogenetic tree. We can thus postulate that these two motifs and the intracellular trafficking they regulate must be essential for efficient replication and propagation of these viruses. We and others have shown that, regardless of whether they are from the oncoretrovirus or the lentivirus family, all retrovirus envelope glycoproteins follow complex intracellular routes [5,8-10,12]. It has been proposed that these different intracellular trafficking routes were part of mechanisms allowing the virus to escape the host immune response by limiting the amount of antigenic envelope glycoproteins at the cell surface of infected cells. Thus, a simian immunodeficiency virus bearing a mutation in the tyrosine-based endocytosis signal of its envelope glycoproteins is attenuated *in vivo *[37], although envelope incorporation into virions and virions infectivity are both normal *in vitro *[38]. Moreover, mutation of the same tyrosine-based endocytosis signal in HIV enhances the immunogenicity of a vaccine preparation, in correlation with enhanced surface expression of the protein [39]. However, the fact that the intracellular trafficking pathways we describe are much more complex than just endocytosis suggests that they play other roles than just limiting the amount of envelope glycoproteins present at the cell surface.

In the last few years, it was demonstrated that the Gag polyproteins of retroviruses hijack various host proteins and use them for assembly and budding of particles (reviewed in [2,3]). All the cellular factors described so far to participate in this phenomenon are proteins that function in different stages of the endosomal trafficking: Nedd4 [40-42], Tsg101 and other ESCRT-1 components [40,42-44], AIP1/ALIX [45], AP2 [46] and AP3 [47]. Growing number of evidence indicate that Gag proteins are transported along the endosomal pathway prior to assembly and budding [42,47-56]. Thus, targeting envelope glycoproteins in the endosomal pathway might help Env encountering Gag and being subsequently incorporated in the nascent virion. That Env glycoproteins constantly traffic in a cycling pathway between TGN and endosomes as we described here would furthermore allow them to wait inside the cell until they encounter the Gag proteins in endosomes, and are subsequently rerouted to be incorporated into budding particles.

Supporting this hypothesis, it has been shown that HIV-1 envelope glycoproteins also follow an anterograde/retrograde pathway and that this trafficking step is required for optimal incorporation of Env into virions and subsequent infectivity of the virus [9]. We also found that bovine leukemia virus envelope cytoplasmic tail possesses dileucine- and tyrosine-based motifs that drive its trafficking in a TGN-endosome cycling pathway (Blot et al, unpublished data). Finally, the tyrosine-based motifs in position 23 in the cytoplasmic tail of MLV and MPMV envelope glycoproteins, which we found are necessary to maintain the protein in the endosomes to the TGN retrograde route, are also necessary for efficient incorporation of Env into virus particles [57,58].

Finally, regulated Env intracellular trafficking might also be important for intracellular Gag sorting and subsequent efficient virus release. Indeed, it has been shown that Env can influence Gag intracellular localization for both MLV [53] and MPMV [48]. MLV can be released in a polarized manner and this process depends on the integrity of the tyrosine-based motif in Env CT [10,59]. MPMV follows the Type-D assembly pathway in which Gag pre-assembled in the cytoplasm. It has been recently shown that pre-assembled MPMV Gag are localized on pericentriolar microdomains and Env is required to promote Gag transport out of this perinuclear site [48]. It will thus be of great interests to test the importance of Env tyrosine-based and dileucine-based motifs in this process. It was long thought that the whole process of retrovirus assembly occurs at the plasma membrane of infected cells. Accumulating evidence now complicates this simple scheme and suggests that retroviruses developed strategies ensuring the specific sorting of their structural proteins into intracellular compartments. These complex routes may be viewed as a funnel, concentrating the different structural components of the viruses from their synthesis sites dispersed throughout the cell towards a unique platform of assembly. The discovery of such mechanisms may provide new targets to develop antiretroviral drugs. Understanding the precise mechanisms that underlie the transport of viral proteins inside the cells and their interactions with host cell factors during assembly and budding appears then as an important future challenge for retrovirology.

## Conclusion

We found here that two unrelated retroviruses, MLV and MPMV, share the capacity to acutely regulate the trafficking of their envelope glycoprotein inside the cells. Env intracellular trafficking involves a cycling loop between the TGN and endosomes. Due to the presence of dileucine- and tyrosine-based motifs conserved in sequence and position in MLV and MPMV Env cytoplasmic tails, Env interact with clathrin adaptors. Thus, both structural Gag and Env proteins hijack the host cell machinery involved in trafficking in the endosomal pathway, which could be used as an assembly platform.

## Methods

### Plasmids and cells

The CD25, CD25-TFR, CD25-MLV and CD25-MPMV chimeras were previously described [12]. Mutagenesis was performed by PCR using the Quickchange™ Site-directed mutagenesis kit (Stratagene) according to the manufacturer's instructions and plasmids were then sequenced by automatic sequencing (sequencing core facility, Institut Cochin, Paris, France). The amino acid sequences of the resulting chimeric proteins are shown in Fig. 2B.

HeLa cells were grown in Dulbecco's Modified Eagle Medium (DMEM) supplemented with 10% fetal calf serum (FCS), gentamycine and 2 mM L-glutamine. Transient transfections were performed using the calcium phosphate procedure. For indirect immunofluorescence assays, 2.10^4 ^cells plated per well of 24-well plates were transfected with 300 ng (steady state analysis) or 500 ng (antibody uptake assays) plasmid. The total quantity of DNA was normalized to 1 μg by adding empty vector (pcDNA3). For flow cytometry analysis, 7.10^5 ^HeLa cells plated in 100 mm dishes were co-transfected with 4 μg of chimera encoding plasmid and 2 μg of pEGFP1 vector (Clontech), which allowed the detection of transfected cells by the expression of green-fluorescent protein (GFP). The total amount of DNA was normalized to 10 μg by adding empty vector.

### Antibodies and Fluorescent Reagents

The 7G7B6 and 2A3A1H monoclonal antibodies (MAb) directed to CD25 were obtained from ascites fluids (gift of A. Dautry-Varsat, Institut Pasteur, Paris, France). The anti-MPR46 is an affinity purified rabbit serum provided by S. Höning (University of Göttingen, Germany)[22]. The anti-Lamp 1 MAb coupled to FITC was purchased from Pharmingen (San Diego CA, USA). The transferrin receptor was revealed using cyanine 3-conjugated human transferrin (gift of A. Dautry-Varsat, Institut Pasteur, Paris, France). The FITC-conjugated dextran was purchased from Molecular Probes.

### Intracellular staining and confocal microscopy

Forty-eight hours after transfection, cells grown on glass coverslips (Polylabo, France) were treated with cycloheximide (500 μM) (Sigma) for 3 h before fixation for 15 min in PBS-4% paraformaldehyde at room temperature, and quenching for 15 min in PBS-0.1 M glycine. Cells were then permeabilized for 40 min with PBS containing 0.05% saponin and 0.2% bovine serum albumin (BSA) (permeabilizing buffer). CD25 chimeras and human MPR46 marker were co-stained using the anti-CD25 7G7B6 MAb (ascites fluid, 1/500 dilution in permeabilizing buffer) and anti-MPR46 affinity purified rabbit antiserum (1/500) for 1 h. After washes, the staining was revealed using FITC-coupled goat anti-mouse Ig and cyanine 3-coupled goat anti-rabbit Ig (Jackson Immunoresearch Laboratories, Inc., 1/300). Cells were mounted in mowiol (Calbiochem, California, USA) and examined under a confocal microscope (MRC-1024, Bio Rad). All images presented are single slices from median sections of cells.

For colocalization with Lamp-1, cells were saturated using non immune murine sera (1/100, in permeabilizing buffer) following the CD25 staining. Lamp-1 was then revealed by a 1 h incubation with 1/50 dilution of FITC-conjugated anti-lamp-1 MAb (Pharmingen, CA, USA) in permeabilizing buffer complemented with a 1/50 dilution of non-immune murine sera. Late endosomal compartments were revealed by incubating living transfected cells with FITC-conjugated dextran (2 mg/ml in complete medium) for 30 min at 37°C followed by a 30 min chase using complete medium, whereas early/recycling compartments were stained after a 30 min internalization of cyanin3-conjugated transferrin (100 nM in serum free medium). Cells were then fixed and CD25 chimeras were revealed as described above.

### Flow cytometry

Cells were collected 48 hours after transfection by incubation with PBS containing 5 mM EDTA for 10 min, pelleted and suspended in ice-cold PBS. They were then incubated for 1 h with the anti-CD25 2A3A1H MAb (ascites fluid, 1/2000) in 100 μl of PBS at 4°C, washed 2× with chilled PBS, and stained with phycoerythrine conjugated goat anti-mouse Ig (Caltag, California, USA) for 1 h at 4°C. The cells were washed and fixed in PBS containing 2% formaldehyde (FAD), and analyzed by flow cytometry after gating on the GFP-positive population.

### Internalization assay

Transfected cells were collected as described above, incubated with the anti-CD25 2A3A1H MAb (ascites fluid, 1/2000) for 1 h on ice, and washed in chilled PBS. Cells were then either kept at 4°C (t = 0) or shifted to 37°C for 30 min, rapidly cooled to 4°C and washed once (t = 30). MAbs bound to the cell surface were then revealed by incubation with phycoerythrine-coupled goat anti-mouse Ig for 1 h at 4°C. After two washes with chilled PBS, cells were fixed in PBS-2% FAD and analyzed by flow cytometry. GFP-negative cells were excluded from the analysis. The internalization of chimeras was estimated as follow: [(mfi_t = 0_)-(mfi_t = 30_)]/[(mfi_t = 0_)-(mfi_neg_)] × 100, where mfi_t _is the mean fluorescence intensity of cells harvested after incubation for either 0 min or 30 min at 37°C and mfi_neg _is the background staining without primary antibody.

### Analysis of the retrograde transport

For analysis of the endosome-to-TGN retrograde transport, HeLa cells were transiently transfected with 500 ng chimera expressing vectors and analyzed 48 h after transfection. Cells were treated 2 h with cycloheximide (500 μM) and incubated with the 7G7B6 MAb (1/500 in chilled PBS) for 1 h on ice. Then cells were shifted at 37°C in complete medium containing cycloheximide for 1 h, fixed and permeabilized as described above. The TGN compartment was revealed by anti-MPR46 affinity purified rabbit serum (1/500), followed by a co-incubation of FITC conjugate anti-mouse Ig (1/300) and cyanine 3 conjugate anti-rabbit Ig (1/300).

### Yeast two-hybrid assays

DNA fragment encoding MLV cytoplasmic tail (amino acid residues 1 to 33; Figure 2A) was generated by PCR and cloned in frame with the LexA binding domain (BD) into the pFBL2-3 vector, a gift of J. Camonis (Institut Curie, Paris), (pFBL-MLV). Point mutations of the tyrosine 23 and leucine 3 residues were introduced by PCR-based site-directed mutagenesis using the appropriate primers to generate the following constructs: pFBL-MLV-L3S, pFBL-MLV-Y23S. Mutations were verified by DNA sequencing. Plasmids for expressing the μ1, γ and β1 chains of AP1 complex, the μ2, α and β2 chains of AP2 and the μ3, δ and β 3 chains of AP3 fused to the Gal4 activation domain (AD) in the pACTII vector were kindly provided by J. S. Bonifacino (NIH, Bethesda, MD) [7] and M. Robinson (University of Cambridge, Cambridge) [60]. The yeast reporter strain L40 containing the HIS3 LexA were co-transformed with the indicated LexA BD and Gal4 AD expression vectors, and plated on selective medium lacking tryptophan and leucine. Double transformants were patched on the same medium and then analyzed for histidine auxotrophy by replica-plating on selective medium lacking tryptophan, leucine and histidine [5].

### GST-pull down assays

DNA fragment containing the cytoplasmic tail of MLV was obtained by PCR and cloned in-frame with GST (glutathione S-transferase) into the pGex-2TH vector to generate pGex-MLV. Point mutations of the essential L3 and Y23 residues were introduced by PCR and the following constructs were obtained: pGex-MLV-L3S, pGex-MLV-Y23S.

Bacterially-expressed GST chimeric proteins and unfused GST (control) were purified and immobilized on GSH-agarose beads as previously described [5]. Coomassie blue staining of polyacrylamide gel was used to control that the beads were coated with the same amount of GST recombinant proteins. GST-fusion proteins (5 μg) immobilized on GSH-agarose beads were incubated 1h at 4°C in PBS containing 2 mg/ml BSA and 0.05% Tween. HeLa cells were lysed in lysis buffer (50 mM Tris pH 8, 150 mM NaCl, 5 mM EDTA, 1% Triton X-100). HeLa cell lysates corresponding to 2.5.10^7 ^cells were incubated overnight at 4°C with 5 μg GST fusion proteins or GST control immobilized on GSH-agarose beads. The beads were then washed five times with lysis buffer. Bound proteins were eluted, separated by SDS-PAGE and revealed by Western blotting with anti-γ adaptin mAb (Transduction laboratories), anti-α adaptin mAb (clone 100/2, Sigma) and anti-δ adaptin mAb (Transduction laboratories).

## Abbreviations

MLV: Moloney murine leukemia virus; MPMV: Mason-Pfizer monkey virus; HIV: human immunodeficiency virus; Env: envelope glycoprotein; CT: cytoplasmic tail; AP: adaptor protein; TGN: Trans Golgi network

## Competing interests

The author(s) declare that they have no competing interests.

## Authors' contributions

VB and MPG designed and conducted the study, performed the experiments and wrote the manuscript. MB helped setting up the assays. CBT contributed to draft the manuscript and conducted the yeast two-hybrid and GST pull down studies. SLV performed the yeast two-hybrid and GST pull down experiments, and contributed to draft the manuscript. CP contributed to the data interpretation and to draft the manuscript.
